# Combination drug screen identifies synergistic drug interaction of BCL-XL and class I histone deacetylase inhibitors in *MYC*-amplified medulloblastoma cells

**DOI:** 10.1007/s11060-023-04526-w

**Published:** 2024-01-07

**Authors:** Simon Zeuner, Johanna Vollmer, Romain Sigaud, Sina Oppermann, Heike Peterziel, Dina ElHarouni, Ina Oehme, Olaf Witt, Till Milde, Jonas Ecker

**Affiliations:** 1 Hopp Children’s Cancer Center Heidelberg (KiTZ), Im Neuenheimer Feld 430, 69120 Heidelberg, Germany; 2 National Center for Tumor Diseases (NCT), NCT Heidelberg, a partnership between DKFZ and Heidelberg University Hospital, Im Neuenheimer Feld 460, 69120 Heidelberg, Germany; 3 Clinical Cooperation Unit Pediatric Oncology, German Cancer Research Center (DKFZ) and German Consortium for Translational Cancer Research (DKTK), Im Neuenheimer Feld 280, 69120 Heidelberg, Germany; 4 Department of Pediatric Hematology and Oncology, Heidelberg University Hospital, Im Neuenheimer Feld 430, 69120 Heidelberg, Germany; 5 Department of Bioinformatics and Omics Data Analytics, German Cancer Research Center (DKFZ), Heidelberg, Germany

**Keywords:** Drug screen, Medulloblastoma, MYC, HDAC, BCL-XL

## Abstract

**Purpose:**

Patients with *MYC*-amplified Group 3 medulloblastoma (MB) (subtype II) show poor progression-free survival rates. Class I histone deacetylase inhibitors (HDACi) are highly effective for the treatment of *MYC*-amplified MB in vitro and in vivo. Drug combination regimens including class I HDACi may represent an urgently needed novel treatment approach for this high risk disease.

**Methods:**

A medium-throughput in vitro combination drug screen was performed in three *MYC*-amplified and one non-*MYC*-amplified MB cell line testing 75 clinically relevant drugs alone and in combination with entinostat. The drug sensitivity score (DSS) was calculated based on metabolic inhibition quantified by CellTiter-Glo. The six top synergistic combination hits were evaluated in a 5 × 5 combination matrix and a seven-ray design. Synergy was validated and characterized by cell counts, caspase-3-like-activity and poly-(ADP-ribose)-polymerase-(PARP)-cleavage. On-target activity of drugs was validated by immunoprecipitation and western blot. BCL-XL dependency of the observed effect was explored with siRNA mediated knockdown of *BCL2L1*, and selective inhibition with targeted compounds (A-1331852, A-1155463).

**Results:**

20/75 drugs effectively reduced metabolic activity in combination with entinostat in all three *MYC*-amplified cell lines (DSS ≥ 10). The combination entinostat and navitoclax showed the strongest synergistic interaction across all *MYC*-amplified cell lines. siRNA mediated knockdown of *BCL2L1*, as well as targeted inhibition with selective inhibitors showed BCL-XL dependency of the observed effect. Increased cell death was associated with increased caspase-3-like-activity.

**Conclusion:**

Our study identifies the combination of class I HDACi and BCL-XL inhibitors as a potential new approach for the treatment of *MYC*-amplified MB cells.

**Graphical abstract:**

**Figure:**
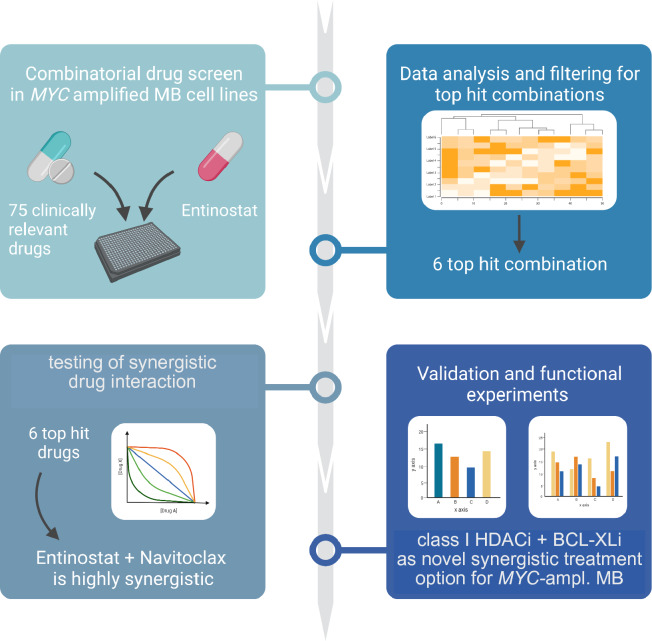
Graphical abstract created with BioRender.com, illustrating the workflow and summarizing main results.

**Supplementary Information:**

The online version contains supplementary material available at 10.1007/s11060-023-04526-w.

## Introduction

Medulloblastoma (MB) is an embryonal brain tumor occurring in children and young adults. The clinical outcome of patients with MB is strongly dependent on the molecular subgroup (WNT, SHH, Group 3 and Group 4) [1, 2]. Group 3 and Group 4 tumors comprise eight molecularly defined subtypes (I-VIII), each displaying distinct cell biology and clinical characteristics [3]. Group 3 MB tumors of subtype II harbor an amplification of the proto-oncogene and transcription factor *MYC* [4]. Patients with *MYC*-amplified MB have a particularly poor prognosis despite intensive conventional treatment regimens [5, 6].

The proto-oncogene *MYC* is overexpressed in the majority of cancers and can potentially affect the expression of all genes [7]. MYC activity amplifies existing transcriptional programs and facilitates cell growth and cell division [8], which explains the strong transforming potential of MYC [9]. To avoid excessive and henceforth potentially tumorigenic MYC activity, MYC is tightly controlled by transcription factor licensing [7]. Furthermore the activity of MYC is directly linked to induction of apoptosis by downregulation of anti-apoptotic and upregulation of pro-apoptotic genes as well as the activation of the caspase-8/t-BID-axis [10]. However, tumor cells can evade MYC-driven apoptosis in a setting of increased pro-survival signaling or in case mutations leading to dysfunctional surveillance mechanisms such as the p53 pathway [10]. Pharmacological interference with apoptosis evasion mechanisms may revert MYC from the main oncogenic driver to the main apoptotic inducer.

We and others have previously shown that class I histone deacetylase (HDAC) inhibition is highly effective for the treatment of *MYC*-amplified MB in vitro and in vivo [11, 12]. The high susceptibility of *MYC-*amplified MB cells to this treatment is explained in part by inhibition of HDAC2 in the HDAC2-MYC-protein complex leading to an impairment of the function of MYC as a transcription factor [7]. Since HDACi have also been described to alter the expression of genes involved in apoptosis it may be that the cytotoxic effect of HDACi in *MYC*-amplified MB is partially based on the reinstitution of apoptosis as a cellular response to *MYC* overexpression [13].

HDACi have been used for the treatment of different cancers for more than a decade. Currently four HDAC inhibitors are FDA-approved for oncological indications [14]. However, only a minority of solid tumors have shown response to HDACi as monotherapy in clinical trials [15]. It becomes clear that a better understanding of the molecular downstream effects of HDAC inhibition, as well as the development of predictive biomarkers for patient selection is crucial to translate promising preclinical findings [16]. Additionally, it is important to consider that aggressive cancers such as *MYC*-amplified Group 3 MB have a strong capacity to develop therapy evasion mechanisms and resistance when confronted with highly cytotoxic multimodal chemotherapy [17]. In line with this observation, single agent targeted treatment approaches are not likely to induce long term responses in high-grade solid tumors [18]. Conversely, combining different targeted agents has shown high potential to reduce the likelihood of resistance development, to increase treatment efficacy by targeting multiple pathways at a time and to reduce side effects [19].

In this study we perform a combination drug screen to identify novel synergistic combination therapies with the class I HDACi entinostat for the treatment of *MYC-*amplified MB.

## Material and methods

### Cell culture and cell lines

The MB cell lines MED8A: CVCL_M137 and UW228-2: CVCL_0572 were cultured as previously described [11]. HD-MB03 is a cell line established in our laboratory, culturing conditions are described in [11]. D425: CVCL_1275 cell line was cultured in Modified Improved Minimum Essential Medium (Gibco, A10489-01), supplemented with 10% fetal bovine serum (FCS). Cell lines were authenticated [20], purity was validated [21]. Absence of mycoplasma contamination was checked monthly with Venor®GeM Mycoplasma PCR Detection Kit (Minerva Biolabs, 11- 1250, Germany). HD-MB03 cells, MED8A cells and D425 cells have been originally derived from Group 3 MB tumors, all of them carry a *MYC*-amplification [22]. UW228-2 cells have been originally derived from a SHH MB tumor and carries no *MYC-*amplification [22].

### Drug screening and drug sensitivity score (DSS) calculation

The drug screen and DSS calculation were conducted as previously described [23]. Drugs with a DSS score > 10 were considered effective. The library [24] was dispensed on 384-well plates by the High Throughput Biomedicine core unit (Institute for Molecular Medicine Finland, HiLIFE, University of Helsinki, Finland), each drug in five different concentrations (in technical duplicates) on a 10,000-fold concentration range. Cells were seeded at a density of 500 cells/well. For the combination screen, a cell line specific EC25 of entinostat (Suppl. Table 1, Suppl. Figure 1A) was added using a Tecan D300e Digital Dispenser (Tecan Life Sciences, Männedorf, Schweiz). After 72h cell viability was measured with CellTiter-Glo®2.0 Cell Viability Assay (Promega, G9243) (CTG) on FLUOstar OPTIMA automated plate reader (BMG Labtech).

### Filtering for the top hit combinations

Three filtering steps were applied to determine the top hit combinations: (1) DSS_combo_ ≥ 10 all three *MYC*-amplified cell lines to filter for the most potent drugs in combination with entinostat. (2) DSS_combo_—DSS_single_ > 0 in at least 2/3 *MYC*-amplified cell lines to filter for drugs that were enhanced in their effect by the addition of entinostat. (3) DSS_single *MYC* ampl_—DSS_single non ampl_ > 0 in at least 2/3 cell lines to filter for *MYC*-amplified preferential drugs.

### Drugs for synergy validation

Drugs are listed as purchased in Suppl. Table 2.

### Single agent and synergy assessment

For single dose response curves 800 cells/well were seeded in 384-well plates (Greiner, 781098) and treated with a concentration range in a 1/2 log distribution. After 72h metabolic activity was assessed with CTG according to manual instructions. Synergistic drug interaction with entinostat was evaluated in a 5 × 5 matrix design and a ray (fixed-ratio) design as previously described [25] and analyzed using SynergyFinder2.0 web-application (https://synergyfinder.fimm.fi) [26] . Normalized data of the ray design were processed by the DrugCombo1.1.1 package in R [27] to retrieve tau values for each ray.

### Cell count experiments

Cell count experiments were performed as previously described [11]. Combination index (CI) values were calculated using CompuSyn-Software (ComboSyn Inc., Paramus, NJ, USA).

### Western blot (WB)

Westerblot and densitometry analysis was performed as previously described [11]. Quantification of Western blot bands was performed using ImageJ (version 1.52p, Wayne Rasband and co., NIH). For antibodies see Suppl. Table 3.

### Immunoprecipitation

Cells were treated with 2500nM navitoclax or solvent control (DMSO) for 6h. Immunoprecipitation (IP) was performed with Dynabeads Protein G Immunoprecipitation Kit (Invitrogen, 10007D) according to manufacturer’s instructions, antibodies were crosslinked to the beads with dimethyl pimelimidate solution (20mM) in triethanolamine buffer (0.2M). For antibodies see Suppl. Table 3.

### Caspase-activity assay

Cells were treated for 48h. The Caspase-3 Fluorometric Assay Kit (BioVision) was used to quantify caspase-3-like-activity according to manufacturer’s instructions and measured on a FLUOstar OPTIMA automated plate reader (BMG Labtech) for 3h.

### siRNA knockdown

Knockdown in MED8A cells was performed as previously described [11]. For siRNA see Suppl. Table 4.

### Statistical analysis and graphical representation

The primary drug screening was performed in two technical replicates. All other experiments were conducted in at least three biological replicates. Error bars indicate mean ± standard deviation (SD). Graphs were generated with GraphPad Prism 5 software (Version 5.01, GraphPad Software Inc., San Diego, USA) and in RStudio (version 1.3.1073) using the ggplot2 package (version 3.3.3). RStudio (R version 4.0.2) was used for the calculation of ANOVA followed by Bonferroni multiple comparison test, as indicated. GraphPad Prism 5 was used to calculate Student’s t-tests as indicated. DSS heatmap was generated with the Morpheus web application (https://software.broadinstitute.org/morpheus/). Hierarchical clustering was performed with Euclidean distance as similarity metric and complete-linkage clustering. DSS values that could not be calculated were assigned the DSS value 0 based on an individual assessment of the dose response curves. Heatmaps depicting metabolic activity at the maximum plasma concentration (c_max_) and ray design synergy assessment were generated with the ComplexHeatmap [28] package (version 2.4.3.) in RStudio.

### mRNA and protein expression data of primary medulloblastoma samples

Primary MB sample mRNA expression data was derived from a public MB gene expression data set (R2 internal identifier: ps_avgpres_mb500affym223_u133p2 [3]), protein expression data was derived from [29]. Groups were compared using One Way ANOVA and subsequent Bonferroni multiple comparisons testing.

## Results

### Combination drug screen in MB cell lines

**Fig. 1:**
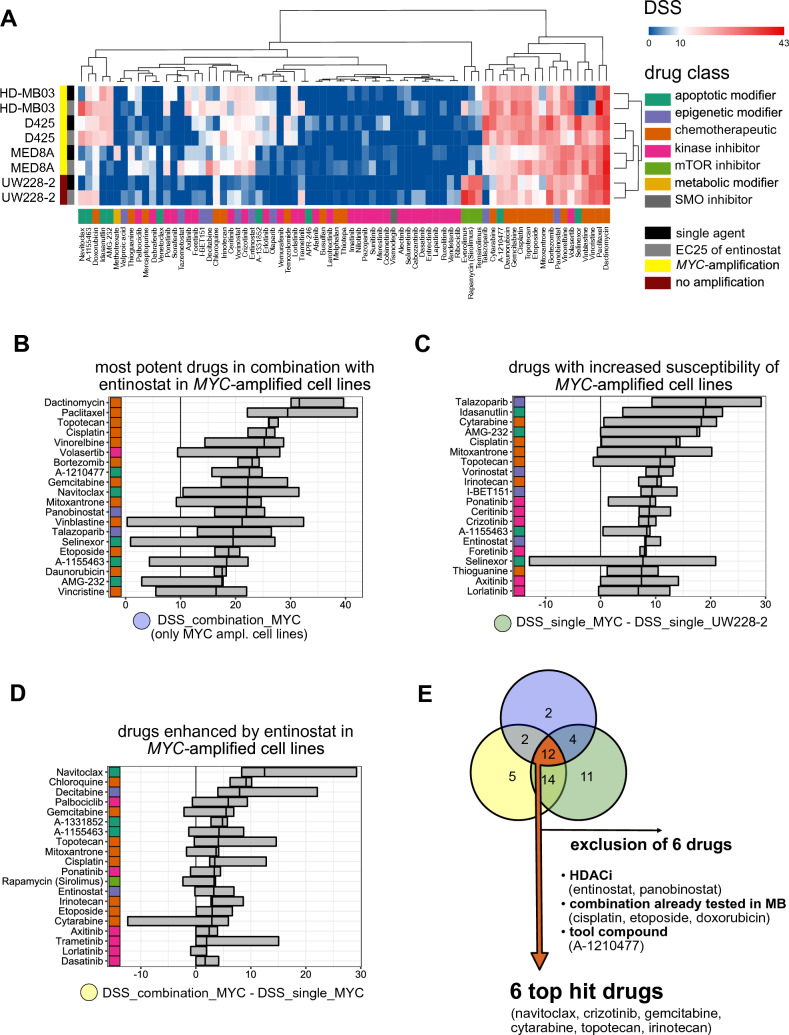
Combination drug screen in MB cell lines. **A** Combination drug screen in four MB cell lines (HD-MB03, D425, MED8A and UW228-2) treated for 72h with five different concentrations of 75 drugs as single agents or in combination with the cell lines specific EC25 of entinostat. Heatmap depicts the drug sensitivity score calculated based on metabolic inhibition. *MYC* amplification status of cell lines, as well as drug class of compounds are color coded as annotated in the legend. *DSS* drug sensitivity score; EC25: ¼ maximal effective concentration. **B**–**D** cross bar plots of DSS scores of drugs in three *MYC*-amplified MB cell lines according to different rankings. Rankings are color coded as shown in the colored circles below the x axis (B = blue, C = green, D = yellow). Grey boxes show range of DSS scores in three *MYC*-amplified cell lines from lowest to highest, the median DSS score is depicted by a vertical black line. Drug class of compounds is color coded as annotated in the legend. Only top 20 drugs of each ranking are shown. **B** DSS score of drugs in combination with EC25 entinostat with DSS > 10 in all *MYC*-amplified MB cell lines. **C** DSS score of single agent drugs with DSS > 0 after subtraction of the DSS of non-*MYC*-amplified cell line UW228-2 in 2/3 *MYC*-amplified MB cell lines. **D** DSS score of drugs in combination with EC25 entinostat > 0 after subtraction of single agent DSS in 2/3 *MYC*-amplified MB cell lines. **E** schematic depiction visualizing the overlap of rankings and applied filter steps to determine top hit drugs for further synergy assessment. *DSS* drug sensitivity score

To identify drugs that are particularly effective for the treatment of *MYC*-amplified MB cells as single agents or in combination with the class I HDACi entinostat, we performed a medium-throughput single agent and combination drug screen with a translational drug library (75 drugs) [24] and the cell line specific EC25 of entinostat in three *MYC*-amplified MB cell lines (HD-MB03, D425, MED8A) and one non-*MYC*-amplified MB cell line (UW228-2) (Fig. 1A). The cell line specific EC25 of entinostat was used for this screening approach to ensure (1) treating cells with a concentration of entinostat that affects cell biology and (2) not to treat cells with a toxic concentration of entinostat to be able to observe a possible additional effect of the combination drug. All cell lines showed distinct drug sensitivity profiles with DSS values ranging from 0 to a maximum of 42.1. Hierarchical clustering separated drug sensitivity profiles of the *MYC*-amplified cell lines from the non-*MYC*-amplified cell line (Fig. 1A). The drug sensitivity profiles of each cell line treated with (combination) or without (single) the EC25 of entinostat clustered together, indicating that the combination treatment did not induce a combination specific drug response pattern across all cell lines. Since entinostat was included in the drug library, response to entinostat treatment could be compared to previously published data to serve as a technical control. All *MYC*-amplified cell lines showed DSS scores > 10 for entinostat single agent treatment indicating strong drug sensitivity. Addition of the EC25 of entinostat (combination) led to stable (HD-MB03) or even increasing (MED8A, D425) DSS scores. In contrast, the non-MYC-amplified cell line UW228-2 showed DSS scores < 3 in both the single agent and combination setting indicating a low drug sensitivity. This is in line with previously published data [11] suggesting technical validity. Next to a variety of conventional chemotherapeutics including the standard of care drug cisplatin (drug class color coded orange), the PLK1-inhibitor volasertib, the pan HDACi panobinostat and the MCL1-inhibitor A-1210477 showed DSS scores > 10 in all tested cell lines irrespective of *MYC*-amplification status (Fig. 1A), pointing towards a strong overall cytotoxicity. Vincristine, the second standard of care drug included in the drug library showed low DSS scores only in the HD-MB03 cell line. Interestingly the non-*MYC*-amplified cell line UW228-2 cell line showed high DSS scores (> 20) for all three tested mTOR inhibitors (everolimus, rapamycin and temsirolimus) indicating a drug class specific effect (Fig. 1A).

Taken together these data show that the combination drug screen is feasible to identify patterns of drug sensitivity comparing cell lines with a different genetic background.

### Identification of combination treatment partners for entinostat

We reasoned that drugs with high potential as combination partners for entinostat for the treatment of *MYC*-amplified MB should meet three criteria: (1) High effectivity: effectively reduce metabolic activity (high DSS) in *MYC*-amplified MB cell lines in combination with entinostat (Fig. 1B). (2) High selectivity: be more effective (higher DSS) in *MYC*-amplified cell lines compared to non-*MYC*-amplified cell (lower DSS) lines (Fig. 1C). (3) Interaction: show enhanced effectivity in combination with entinostat (higher DSS) compared to the single agent (lower DSS) treatment (Fig. 1D).

Based on the intersection of drugs meeting all three criteria, we identified 12 drugs as potentially feasible for a combination therapy (Fig. 1E). Interestingly, these 12 drugs included two HDACi (entinostat and panobinostat), as well as three drugs that have already been shown to synergize with HDACi in MB (cisplatin, etoposide, doxorubicin) [30], indicating robustness of the applied filtering algorithm. After excluding HDACis, the previously described interacting drugs and the pre-clinical compound A-1210477, six novel potential combination partners (navitoclax, crizotinib, topotecan, irinotecan, gemcitabine and cytarabine) remained.

### Navitoclax interacts synergistically with entinostat in *MYC*-amplified MB cell lines

In a next step the identified six candidate drugs needed to be validated as synergistic drug interaction partners of entinostat. First, the single agent dose response curves of all six drugs were determined to calculate each drug’s cell line specific IC50 (Suppl. Figure 1B, Suppl. Figure 2A). The clinical relevance of the single agent drug response was determined by assessment of viability after treatment with the maximal clinically achievable plasma concentration (c_max_) (Suppl. Figure 2B). While gemcitabine and cytarabine reduced metabolic activity at c_max_ independent of *MYC*-amplification status, crizotinib, topotecan, navitoclax and irinotecan reduced the metabolic activity at c_max_ only in the *MYC*-amplified cell lines (Suppl. Figure 2B).

**Fig. 2:**
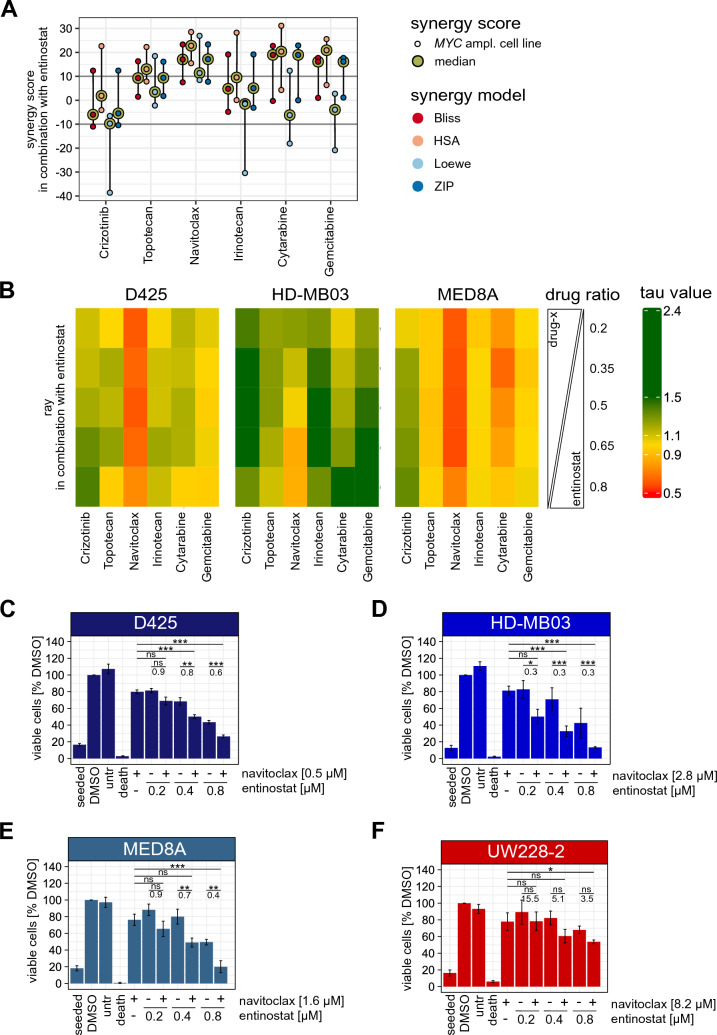
Navitoclax synergizes with entinostat in *MYC*-amplified MB cell lines. **A** Summary plot of matrix design synergy assessment of the top 6 drugs in combination with entinostat in HD-MB03, D425 and MED8A cell lines. Synergy scores are depicted for each cell line (lowest, median, highest) and color coded according to the applied synergy model (Bliss, HSA, Loewe and ZIP) as shown in the figure legend. Values > 10 indicate a synergistic, < 10 to > − 10 additive and < − 10 antagonistic drug interaction. Synergy score of the median cell line is highlighted in light green. **B** heatmaps of the ray design synergy assessment. 5 rays with fixed drug ratios were tested for each cell line and drug combination. Tau values < 0.9 indicate synergistic (red), < 1.1 to > 0.9 additive (yellow) and > 1.1 antagonistic (green) drug interaction for each ray. **C**–**F** Number of viable cells of D425 (**C**), HD-MB03 (**D**), MED8A (**E**) and UW228-2 (**F**) cells treated with a cell line specific EC25 of navitoclax and three increasing concentrations of entinostat as indicated. The number of viable cells are normalized to the DMSO control. Staurosporine was used as positive control. Significant differences were determined by One Way ANOVA and subsequent Bonferroni multiple comparisons testing *p < 0.05; **p < 0.01; ***p < 0.001; ns, not significant. Numbers below lines indicating compared bars describe combination indices (CI) as calculated using the CompuSyn software. CI < 1 indicates synergism, CI = 1 additivity and CI > 1 antagonism on the tested combination. CIs were rounded to one position after the decimal point. Seeded: number of seeded cells; untr: untreated; death: staurosporin death control

To evaluate synergy between entinostat and the six selected compounds in all three *MYC*-amplified cell lines, both a 5 × 5 matrix design and a ray design were used as complementary methods. The combination of entinostat and navitoclax showed a median synergy score > 10 for all three *MYC*-amplified cell lines across all four tested models (Bliss, HSA, Loewe, ZIP) in the matrix design (Fig. 2A), indicating a synergistic drug interaction. The other five tested combinations showed indications for an overall additive behavior with mean synergy scores between + 10 and − 10 (Fig. 2A; Supplementary Figs. 3–8).

Similarly, in the ray design, only the combination of entinostat and navitoclax showed synergistic rays (tau value < 0.9) in all three *MYC*-amplified cell lines (Fig. 2B). While in the D425 and MED8A cell lines each of the five tested rays showed a synergistic drug interaction, in HD-MB03 cells only the two rays with the highest concentration ratio of entinostat/navitoclax showed indications for synergy (Fig. 2B). Synergistic drug interaction of entinostat and navitoclax was validated by determination of number of viable cells after combination treatment. A synergistic drug interaction (CI value < 1.0) was observed in 9/9 tested conditions in all three *MYC*-amplified cell lines (Fig. 2C–E), while no synergy was observed in the non-*MYC*-amplified cell line UW228-2 (Fig. 2F).

In summary, the combination of entinostat and navitoclax showed a synergistic drug interaction in both synergy assessments, metabolic activity and cell counts, across all *MYC-*amplified cell lines. Importantly, synergistic interaction of entinostat and navitoclax was observed at clinically relevant concentrations (Suppl. Figure 1, Suppl. Figure 3).

### Synergism of combined class I HDACi and BCL-XLi is a drug class effect

**Fig. 3:**
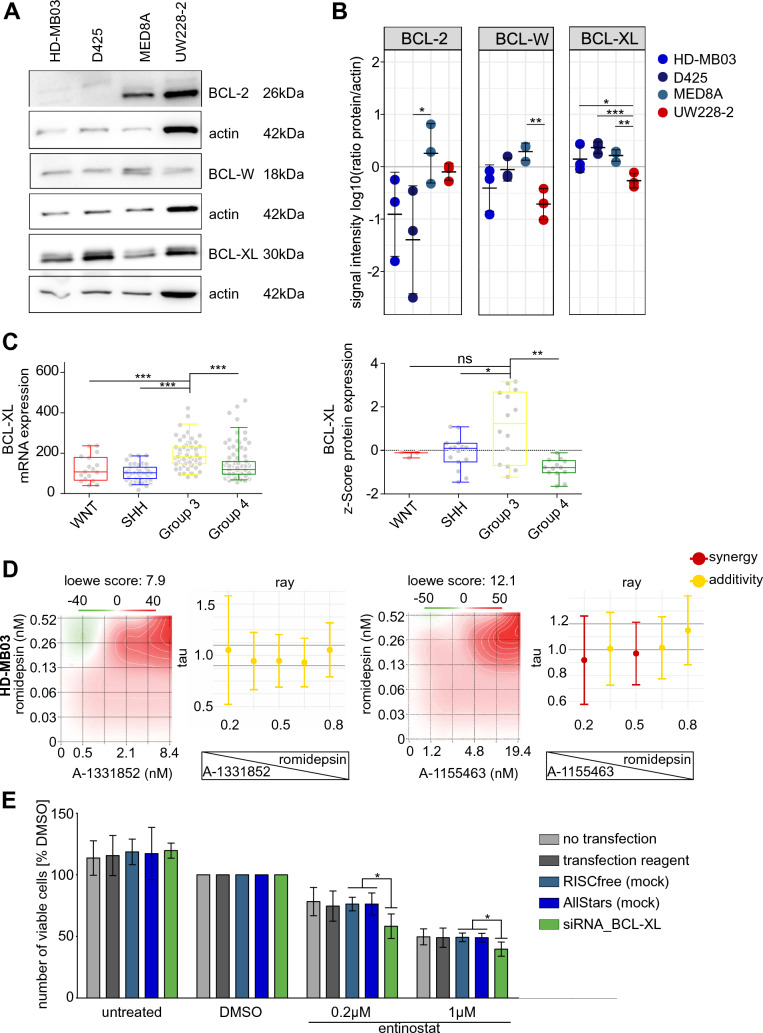
Validation of BCL-XL as relevant drug target in combination with class I HDACi. **A** Target expression validation by western blot of BCL2, BCL-W and BCL-XL in untreated HD-MB03, D425, MED8A and UW228-2 cell line samples. **B** Densitometric quantification of BCL-2, BCL-W and BCL-XL in relation to actin signal intensity in three independent experiments. Significant differences were determined by One Way ANOVA and subsequent Bonferroni multiple comparisons testing *p < 0.05; **p < 0.01; ***p < 0.001. **C** Box-dot plots comparing *BCL2L1* mRNA expression (left) and protein expression (right) in primary MB samples. Dots represent single samples. Boxes extend from 25 to 75th percentiles, whiskers extend from 5 to 95th percentiles, lines at median. *p < 0.05, **p < 0.01; ***p < 0.001; ^ns^ not significant (one-way ANOVA with Bonferroni's Multiple Comparison Test). **D** matrix design (heatmap) and ray design (crossbar plot) synergy assessment of romidepsin and A-1331852 (left) and A-1155463 (right) in HD-MB03 cells. Heatmap depicts loewe score as calculated by synergy finder (SynergyFinder2.0) in the matrix design with the indicated concentrations. Cross bar plots depict mean tau values with standard deviation in five different rays from three independent experiments, concentration ratio of rays indicated on x axis and visualized below x axis. Interpretation of tau values color coded as indicated in figure legend. **E** number of viable cells in percentage of DMSO control after siRNA mediated knockdown of BCL-XL (48h) and treatment with entinostat (72h). Transfection conditions (controls and siRNA knockdown) are color coded as indicated in the figure legend. Bars depict mean value of three independent experiments, standard deviation is indicated. Significant differences were determined by student’s T-test *p < 0.05

Navitoclax effectively inhibits three members of the BCL2-protein family: BCL-2, BCL-W and BCL-XL. Target presence analysis in the investigated cell lines revealed expression of BCL-XL and BCL-W in all four tested cell lines (Fig. 3A), with higher BCL-XL expression in all *MYC*-amplified cell lines compared to the non-*MYC*-amplified cell line UW228-2 (Fig. 3B). BCL-2 protein was not detectable in the two *MYC*-amplified cell lines HD-MB03 and D425 (Fig. 3A, B) suggesting that BCL-2 does not play a major part in the observed response of these cell lines to navitoclax treatment. In line with this observation, treatment with the BCL-2 specific inhibitor venetoclax in the drug screening experiments did not induce any relevant cytotoxicity in the treated cell lines (Fig. 1A). Analysis of publicly available mRNA expression data[3] as well as protein expression data [29] in primary MB tumor samples revealed mRNA and protein expression of BCL-2, BCL-W and BCL-XL in all four molecular groups of MB (Fig. 3C [BCL-XL], Suppl. Figure 9A, B [BCL-2, BCL-W]). Only BCL-XL is expressed significantly higher in Group 3 MB compared to all other MB groups (Fig. 3C) both on mRNA and protein level. BCL-2 is significantly higher expressed in SHH MB compared to all other groups on both mRNA and protein level (Suppl. Figure 9A, B). BCL-W shows no significant differential expression between groups on mRNA level, while it is significantly higher expressed in Group 3 compared to Group 4 tumors on protein level (Suppl. Figure 9A, B). Within Group 3/4 or Group 3, respectively, no correlation with subtypes I-VIII or *MYC* expression levels was observed (Suppl. Figure 10A, B). Taken together with the previous results, these data point towards BCL-XL, and not BCL-2, as the most relevant target of navitoclax in *MYC*-amplified MB cells.

Concurrent with this hypothesis, while not particularly effective as single agents (Fig. 1A), both BCL-XL-specific inhibitors A-1331852 and A-1155463 included in the translational drug library ranked within the top 7 drugs in addition to navitoclax with regard to effectiveness in interaction with entinostat (DSS_combo − DSS_single) (Fig. 1D). To confirm that the observed synergy between class I HDAC inhibitors and BCL-XL inhibitors is a drug class specific effect, a synergy assessment of A-1331852 and A-1155463 in combination with the FDA approved HDAC1/2 inhibitor romidepsin was performed in HD-MB03 and D425 cells, also showing additive to synergistic drug interaction (Fig. 3D, Suppl. Figure 10C). To validate BCL-XL as a relevant target for the combination treatment with entinostat, we performed siRNA mediated knockdown of the BCL-XL gene *BCL2L1* in *MYC*-amplified MED8A cells in combination with entinostat treatment. Knockdown of BCL-XL with two siRNAs led to significantly reduced levels of BCL-XL after 48h (Suppl. Figure 10D). In line with the low drug sensitivity score of single agent BCL-XL specific inhibitors in the drug screen (Fig. 1A) the knockdown of BCL-XL alone did not reduce the number of viable cells. However, additional treatment with entinostat for 72h significantly reduced the number of viable cells compared to entinostat treatment alone (Fig. 3E).

Taken together these data show that simultaneous inhibition of class I HDACs and BCL-XL is an effective and synergistic drug combination for the treatment of *MYC*-amplified MB cells.

### On target activity and induction of apoptosis

**Fig. 4:**
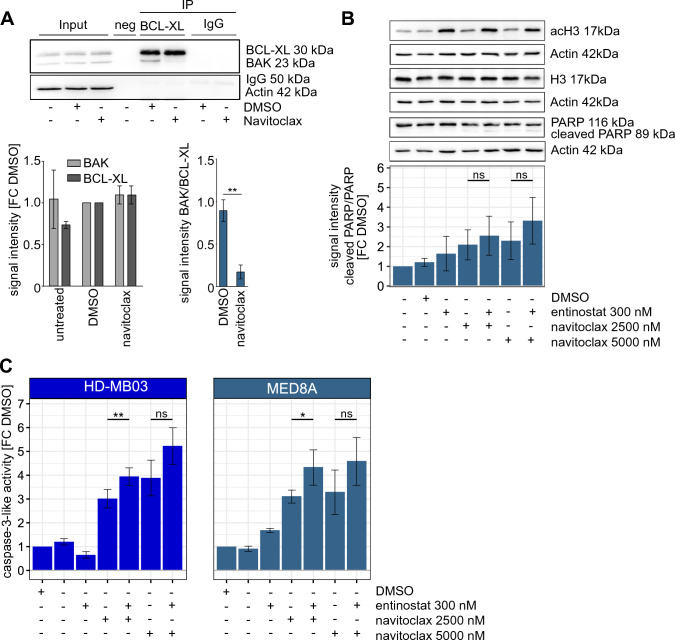
On target activity and induction caspase-3-like-activity. **A** Western blot of IP samples of HD-MB03 cells treated with 2500nM navitoclax or solvent control for 6h. Pull down of BCL-XL and detection of BAK, BCL-XL and actin. Respective densitometric quantification of BAK and BCL-XL signal intensity in treated and untreated input samples (relative to DMSO), as well as ratio of BAK to BCL-XL in IP samples of 3 independent experiments is shown below. Unpaired T-test: **p < 0.01. **B** Western blot analysis of MED8A cells treated with entinostat and navitoclax or the combination at indicated concentrations for 24h. Detection of PARP, cleaved PARP, AcH3, H3 and actin. Respective densitometric quantification of the ratio of cleaved PARP and uncleaved PARP as FC of untreated shown below. Unpaired T-test: ns: not significant. **C** Bar charts of caspase-3-like activity as fold change (FC) of DMSO in HD-MB03 and MED8A cells treated with entinostat and navitoclax for 48h. Unpaired T-test: *p < 0.05; **p < 0.01; *ns* not significant, *FC* fold change

To validate the on-target activity of navitoclax in *MYC*-amplified MB cells, we performed immunoprecipitation (IP) of BCL-XL in HD-MB03 cells, best representative of primary *MYC*-amplified Group 3 MB tumors [31]. Cells were treated for 6h with navitoclax showing significantly decreased binding of BCL-XL to BCL-2 homologous antagonist/killer (BAK) (Fig. 4A). On-target activity of entinostat was confirmed by hyperacetylation of histone H3 upon single agent treatment with entinostat, as well as in combination with navitoclax in MED8A (Fig. 4B) and HD-MB03 cells (Suppl. Figure 11).

According to the function of BCL-XL as an important anti-apoptotic protein, inhibition of BCL-XL can lead to increased levels of apoptosis. To quantify induction of apoptosis upon single agent and combination treatment, we determined cleavage of PARP protein in HD-MB03 and MED8A cells. In MED8A cells combination treatment showed a trend towards increased cleaved PARP compared to single agent treatment (Fig. 4B). In HD-MB03 cells no trend towards increased PARP cleavage upon combination treatment was observed (Suppl. Figure 11). To further evaluate a possible induction of apoptosis, we conducted a caspase-3-like activity assay of HD-MB03 and MED8A cells treated for 48h with entinostat and navitoclax as single agents and in combination, showing a dose dependent trend towards or significant increase of caspase-3-like activity in combination conditions compared to single agent treatment (Fig. 4C).

Taken together these data show on-target activity of both entinostat and navitoclax and that this co-treatment leads to increased caspase-3-like activity in *MYC*-amplified MB cells.

## Discussion

In the past decade considerable advances have been made in our clinical and molecular understanding of *MYC* driven MB. *MYC-*amplification has been established as a high risk marker and has been successfully implemented into clinical risk stratification strategies [2]. Currently this improved risk stratification is most likely to benefit patients that are now stratified into lower risk groups since they may ultimately benefit from a carefully reduced treatment intensity regimen [32]. However, for the high-risk group of patients with *MYC*-amplified MB it appears unlikely that a more intensive treatment regimen based on chemotherapy and irradiation has the potential to improve overall survival rates [32, 33]. Unfortunately only a few targeted treatment approaches for patients with *MYC*-amplified MB such as entinostat, nivolumab, BMS-986158 and BMS-986378 are currently under clinical investigation (e.g. NCT03838042; NCT03936465).

Combination drug screenings are a very efficient way to identify potentially synergistic drug combinations in vitro [34]. Large scale unbiased combination screens have shown that synergistic drug combinations more often involve targeted compounds, rather than conventional chemotherapy [35, 36]. With a growing number of data from large scale combination drug screens available it also becomes clear that this data has to be interpreted and validated with great caution with regard to the clinical relevance of discovered synergistic drug combinations [35]. In particular, the selection of appropriate model systems, the choice of drugs and drug concentrations are important to increase clinical translatability [35]. In our study the experimental design of the drug screen was set to increase clinical relevance by (1) the use of a translational, i.e. clinically available drug library, by (2) testing of five different concentrations to be able to calculate the drug sensitivity score and by (3) testing of different cell lines with a clear, biomarker-defined molecular background (± *MYC* amplification). This resulted in the identification of 12 drugs that were predicted to be highly effective in combination with entinostat in *MYC*-amplified MB cells. Since only a single dose of entinostat (cell line specific EC25) was tested in combination with the drug library in the primary screen, a secondary screen was performed to quantify the predicted synergy of these drugs of interest. In line with the notion that synergistic drug combinations are relatively rare [36] validation experiments confirmed a truly synergistic drug interaction only for one drug combination, namely the combination of class I HDACi entinostat and BCL-XL inhibitor navitoclax. For the validation experiments, we chose two complementary methods (matrix design and ray design) which uncovered that in HD-MB03 cells the synergistic drug interaction of entinostat and navitoclax was dependent on the ratio of both drugs, underlining the importance of testing different drug ratios to optimize treatment regimens of novel drug combinations [37].

The combination of class I HDACi and BCL-XLi has not been studied in MB, and data in other cancer entities are scarce. Vorinostat (a pan-class HDACi) and navitoclax were shown to have synergistic cytotoxic effects in small lung cancer cell lines leading to increased levels of *NOXA*-dependent apoptosiss [38]. Similarly, vorinostat and navitoclax act synergistically in mantel cell lymphoma cells inducing high levels of apoptosis in combination correlating with a transcriptional activation of pro-apoptotic genes such as *BIM*, *BMF*, and *NOXA* [39]. In line with these results we observed increased levels of caspase-3-like activity in *MYC-*amplified MB cell lines treated with entinostat and navitoclax, which is of particular interest given the pro-apoptotic features of MYC.

*MYC* overexpression itself is a well-established inductor of apoptosis [10], either by the ARF-p53 pathway or by a perturbation of the steady state levels of pro- and anti-apoptotic proteins of the BCL-2 homologous (BH) family [40]. Cancer cells with oncogenic levels of MYC need to establish ways to escape the pro-apoptotic features of MYC, and different examples of apoptosis evasion have been described across entities [41]. For example, by activation of anti-apoptotic proteins such as BCL-2 in leukemia [42] and MCL1 in lymphomas [43], and by p53 silencing with long non-coding RNAs in breast- and lung cancer [44]. Particularly in Group 3 MB it has been shown recently that apoptosis evasion is partially mediated by MYC induced suppression of ARF [45]. We have previously shown that class I HDAC inhibition in *MYC*-amplified MB cells leads to MYC protein accumulation [7].

The here described observation that *MYC*-amplified MB cells are highly susceptible to treatment with a combination of class I HDACis and BCL-inhibitors including BCL-XL in their profile suggests that BCL-XL may play an important role in evasion of *MYC*-induced apoptosis in these cells. This is in line with previously published data describing BCL-XL dependency of *MYC*-amplified MB in vitro and in vivo [46], but also in plasma cell neoplasms [47] and lymphoma [48]. However, it is important to note that knockdown of BCL-XL alone did not induce a significant reduction of viable cells in our data. Previously published data describes an apoptosis sensitizing effect of BCL-XL inhibition in *MYC*-amplified MB cell lines when additionally treated with apoptosis inducing chemotherapeutic agents such as doxorubicin [49]. This suggests that part of the observed synergistic cytotoxicity of class I HDACi and BCL-XLi may be caused by the additional apoptosis inducing effect of class I HDACi.

Class I HDACi have been described to induce apoptosis by various mechanisms, including increased DNA damage caused by excessive euchromatization, ROS formation and impairment of DNA damage repair mechanisms by perturbation of the expression of involved genes [50, 51]. Additionally, we have previously shown that class I HDACi is particularly effective in *MYC*-amplified MB cell lines inducing apoptosis [11] and that entinostat treatment leads to a stabilization and accumulation of transcriptionally inactive MYC protein in the cell [7]. It may be speculated that the HDACi induced accumulation of MYC increases the pro-apoptotic effect of MYC. This is in line with published data showing that transient stabilization of MYC by inhibition of glycogen synthase kinase 3β (GSK-3β), which normally targets MYC for proteasomal degradation, sensitizes leukemia cells to chemotherapy leading to increased apoptotic cell death [52]. Taken together it may well be that both *MYC*-amplification and class I HDACi represent triggers of apoptosis in *MYC*-amplified MB, which is particularly effective when induction of apoptosis is facilitated by concurrent inhibition of BCL-XL.

To our knowledge no data is available on the blood brain barrier penetrance of navitoclax, A-1155463 and A-1331852. Given the relatively high molecular weight of these compounds (navitoclax 974.61 g/mol, A-1155463 669.79 g/mol, A-1331852 658.81 g/mol) a very high BBB penetrance may not be anticipated. However, Group 3 MB tumors usually present with a very strong uptake of contrast agent used in MRI imaging indicating a disrupted blood brain barrier [53]. This suggests that the high molecular weight of BCL-XL targeting agents does not necessarily impede accumulation of effective drug concentrations in these tumors.

## Conclusion

The data presented in this study provide in vitro evidence that class I HDACi and BCL-XLi represent a novel synergistic drug combination for the treatment of *MYC*-amplified MB cells. Further investigations in vivo are warranted to explore the translational potential of this novel drug combination for the treatment of patients with these high risk tumors.

### Supplementary Information

Below is the link to the electronic supplementary material.

Supplementary file1 (PDF 3254 KB)—Supplementary Figure S1: Single treatment dose response curves of entinostat, navitoclax, crizotinib and gemcitabine. A Single treatment dose response curves of entinostat in D425, HD-MB03, MED8A and UW228-2 cell lines determined by metabolic activity assay. Dark grey line depicts the maximum plasma concentration in patients. Light grey area depicts the concentration range used for the synergy assessments with the 6 top hit drugs. B Single treatment dose response curves of navitoclax, crizotinib, gemcitabine in D425, HD-MB03, MED8A and UW228-2 cell lines determined by metabolic activity assays. Dark grey line depicts the maximum plasma concentration in patients. Light grey area depicts the concentration range used for the synergy assessments with the 6 top hit drugs.Supplementary Figure S2: Single treatment dose response curves of cytarabin, topotecan and irinotecan. A Single treatment dose response curves of cytarabin, topotecan and irinotecan in D425, HD-MB03, MED8A and UW228-2 cell lines determined by metabolic activity assays. Dark grey line depicts the maximum plasma concentration in patients. Light grey area depicts the concentration range used for the synergy assessments with the 6 top hit drugs. B Heatmap of metabolic activity at reported maximal plasma concentration (cmax) in patients. D425, HD-MB03, MED8A and UW228-2 cells were treated with concentrations of entinostat, navitoclax, crizotinib, topotecan, irinotecan, cytarabine and gemcitabine on a 1/2 log distribution. Dose response curves were calculated and metabolic activity at the reported cmax was determined. Supplementary Figure S3: Synergy evaluation of entinostat and navitoclax. Synergy assessment with dose response matrix depicting percentage of inhibition (left), heatmap with loewe synergy score (center) and crossbar plot of ray design (right) of entinostat and navitoclax in three MYC-amplified MB cell lines. Dose-response matrix depicts metabolic inhibition in percentage of DMSO control. Heatmap depicts loewe score as calculated by synergy finder (SynergyFinder2.0) in the matrix design with the indicated concentrations. Cross bar plots depict mean tau values with standard deviation in five different rays from three independent experiments, concentration ratio of rays indicated on x axis and visualized below x axis. Interpretation of tau values color coded as indicated in figure legend.Supplementary Figure S4: Synergy evaluation of entinostat and crizotinib. Synergy assessment with dose response matrix depicting percentage of inhibition (left), heatmap with loewe synergy score (center) and crossbar plot of ray design (right) of entinostat and crizotinib in three MYC-amplified MB cell lines. Dose-response matrix depicts metabolic inhibition in percentage of DMSO control. Heatmap depicts loewe score as calculated by synergy finder (SynergyFinder2.0) in the matrix design with the indicated concentrations. Cross bar plots depict mean tau values with standard deviation in five different rays from three independent experiments, concentration ratio of rays indicated on x axis and visualized below x axis. Interpretation of tau values color coded as indicated in figure legend.Supplementary Figure S5: Synergy evaluation of entinostat and gemcitabine. Synergy assessment with dose response matrix depicting percentage of inhibition (left), heatmap with loewe synergy score (center) and crossbar plot of ray design (right) of entinostat and gemcitabine in three MYC-amplified MB cell lines. Dose-response matrix depicts metabolic inhibition in percentage of DMSO control. Heatmap depicts loewe score as calculated by synergy finder (SynergyFinder2.0) in the matrix design with the indicated concentrations. Cross bar plots depict mean tau values with standard deviation in five different rays from three independent experiments, concentration ratio of rays indicated on x axis and visualized below x axis. Interpretation of tau values color coded as indicated in figure legend.Supplementary Figure S6: Synergy evaluation of entinostat and cytarabine. Synergy assessment with dose response matrix depicting percentage of inhibition (left), heatmap with loewe synergy score (center) and crossbar plot of ray design (right) of entinostat and cytarabine in three MYC-amplified MB cell lines. Dose-response matrix depicts metabolic inhibition in percentage of DMSO control. Heatmap depicts loewe score as calculated by synergy finder (SynergyFinder2.0) in the matrix design with the indicated concentrations. Cross bar plots depict mean tau values with standard deviation in five different rays from three independent experiments, concentration ratio of rays indicated on x axis and visualized below x axis. Interpretation of tau values color coded as indicated in figure legend.Supplementary Figure S7: Synergy evaluation of entinostat and topotecan. Synergy assessment with dose response matrix depicting percentage of inhibition (left), heatmap with loewe synergy score (center) and crossbar plot of ray design (right) of entinostat and topotecan in three MYC-amplified MB cell lines. Dose-response matrix depicts metabolic inhibition in percentage of DMSO control. Heatmap depicts loewe score as calculated by synergy finder (SynergyFinder2.0) in the matrix design with the indicated concentrations. Cross bar plots depict mean tau values with standard deviation in five different rays from three independent experiments, concentration ratio of rays indicated on x axis and visualized below x axis. Interpretation of tau values color coded as indicated in figure legend.Supplementary Figure S8: Synergy evaluation of entinostat and irinotecan. Synergy assessment with dose response matrix depicting percentage of inhibition (left), heatmap with loewe synergy score (center) and crossbar plot of ray design (right) of entinostat and irinotecan in three MYC-amplified MB cell lines. Dose-response matrix depicts metabolic inhibition in percentage of DMSO control. Heatmap depicts loewe score as calculated by synergy finder (SynergyFinder2.0) in the matrix design with the indicated concentrations. Cross bar plots depict mean tau values with standard deviation in five different rays from three independent experiments, concentration ratio of rays indicated on x axis and visualized below x axis. Interpretation of tau values color coded as indicated in figure legend.Supplementary Figure S9: Target expression analysis in subgroups. A mRNA expression of BCL-2 (left) and BCL-W (right) in primary MB tumors. B protein expression of BCL-2 (left) and BCL-W (righ) in primary MB tumors. Dots represent single samples. Boxes extend from 25-75th percentiles, whiskers extend from 5-95th percentiles, lines at median. *p < 0.05, **p < 0.01; ***p < 0.001; ns not significant (one-way ANOVA with Bonferroni's Multiple Comparison Test).Supplementary Figure S10: Target expression analysis and validation of BCL-XL as relevant target. A Box-dot plot comparing BCL-XL mRNA expression in Group 3/4 subtypes (I – VIII) in primary MB tumor samples. Dots represent single samples. Boxes extend from 25-75th percentiles, whiskers extend from 5-95th percentiles, differences between groups are not significant (one-way ANOVA with Bonferroni's Multiple Comparison Test). B Correlation analysis of BCL-XL and MYC mRNA expression in primary Group 3 MB revealing no correlation of gene expression. C matrix design (heatmap) and ray design (crossbar plot) synergy assessment of romidepsin and A-1331852 (left) and A-1155463 (right) in D425 cells. Heatmap depicts loewe score as calculated by synergy finder (SynergyFinder2.0) in the matrix design with the indicated concentrations. Cross bar plots depict mean tau values with standard deviation in five different rays from three independent experiments, concentration ratio of rays indicated on x axis and visualized below x axis. Interpretation of tau values color coded as indicated in figure legend. D westernblot analysis of BCL-XL protein expression after 48h of siRNA mediated knockdown of BCL-XL (or mock transfection) in MED8A cells.Supplementary Figure S11: Target protein evaluation of entinostat and navitoclax. Western blot analysis of HD-MB03 cells treated with entinostat and navitoclax or the combination at indicated concentrations for 24h. Detection of PARP, cleaved PARP, AcH3, H3 and actin. Respective densitometric quantification of the ration of cleaved PARP and uncleaved PARP as FC of DMSO shown below. Unpaired T-test: ** p < 0.01.

Supplementary file2 (XLSX 10 KB)

Supplementary file3 (XLSX 10 KB)

Supplementary file4 (XLSX 10 KB)

Supplementary file5 (XLSX 10 KB)

## Data Availability

The datasets generated and analyzed during the current study are available in the supplementary files or from the corresponding author on reasonable request.
