# Supplementary material for: Combination drug screen identifies synergistic drug interaction of BCL-XL and class I histone deacetylase inhibitors in MYC-amplified medulloblastoma cells
Source: J Neurooncol. 2024 Jan 7;166(1):99–112. doi: 10.1007/s11060-023-04526-w (PMC10824805; doi:10.1007/s11060-023-04526-w)

Supplementary Figure 1

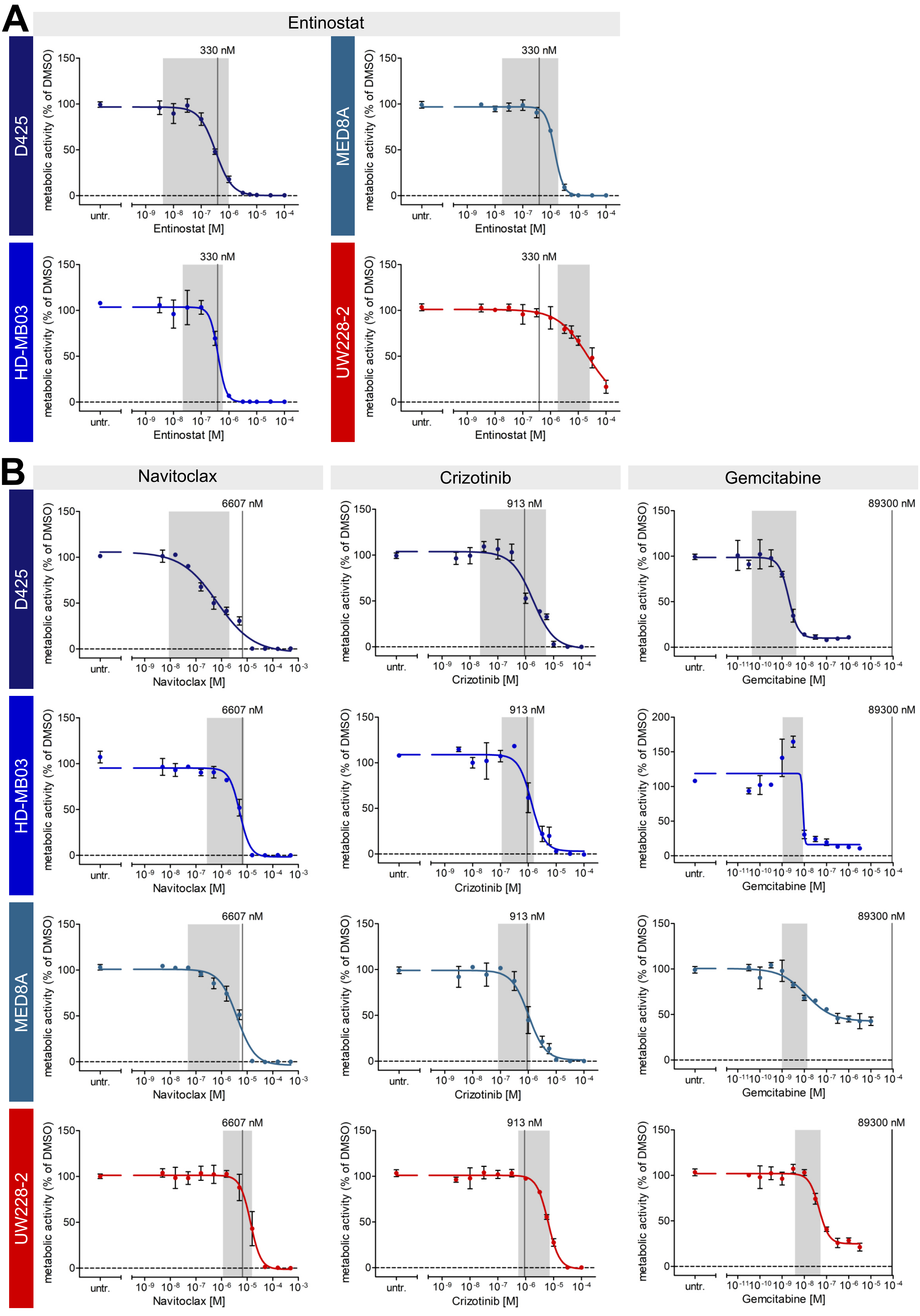

Supplementary Figure 2

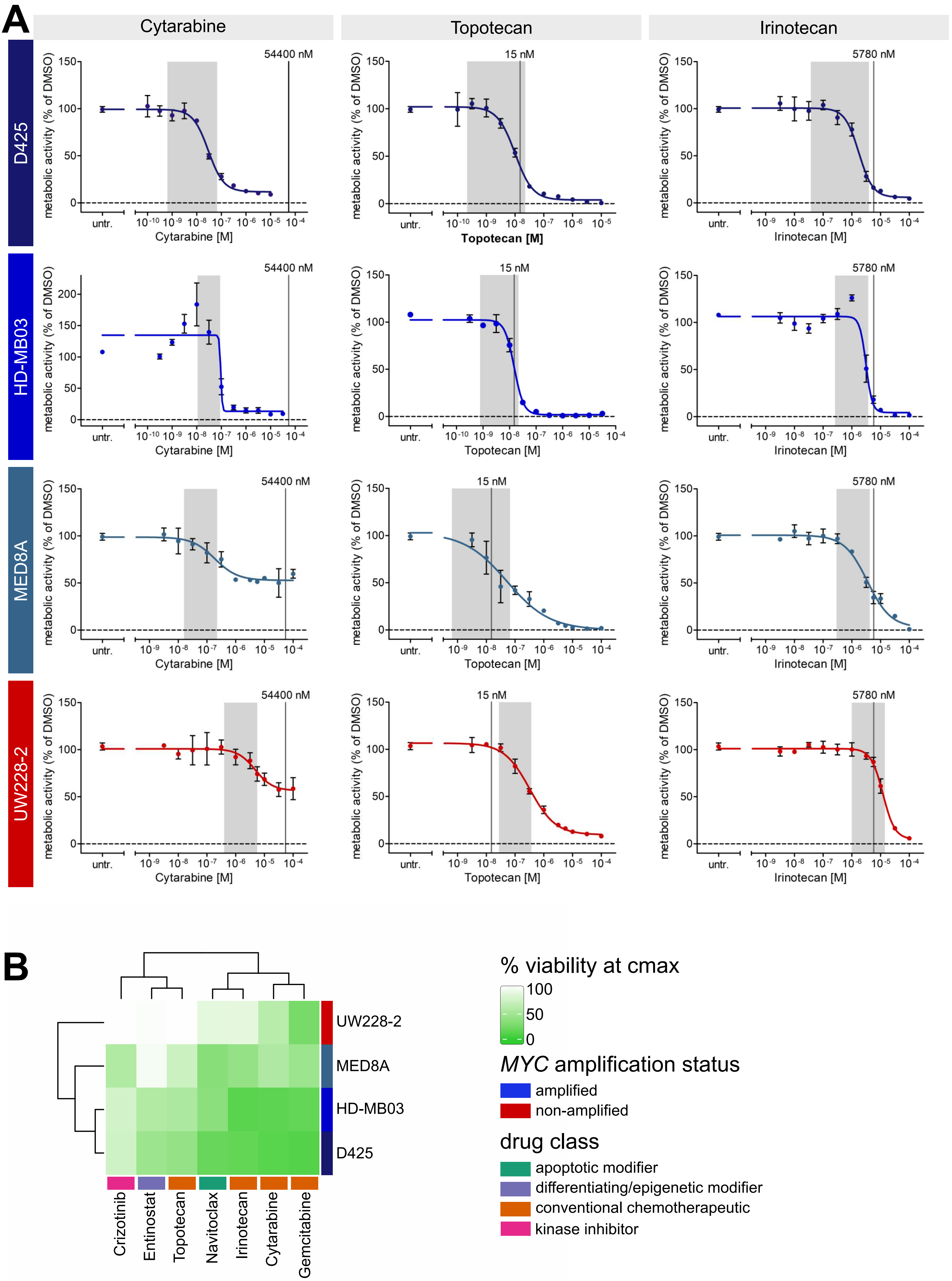

Supplemantary Figure 3

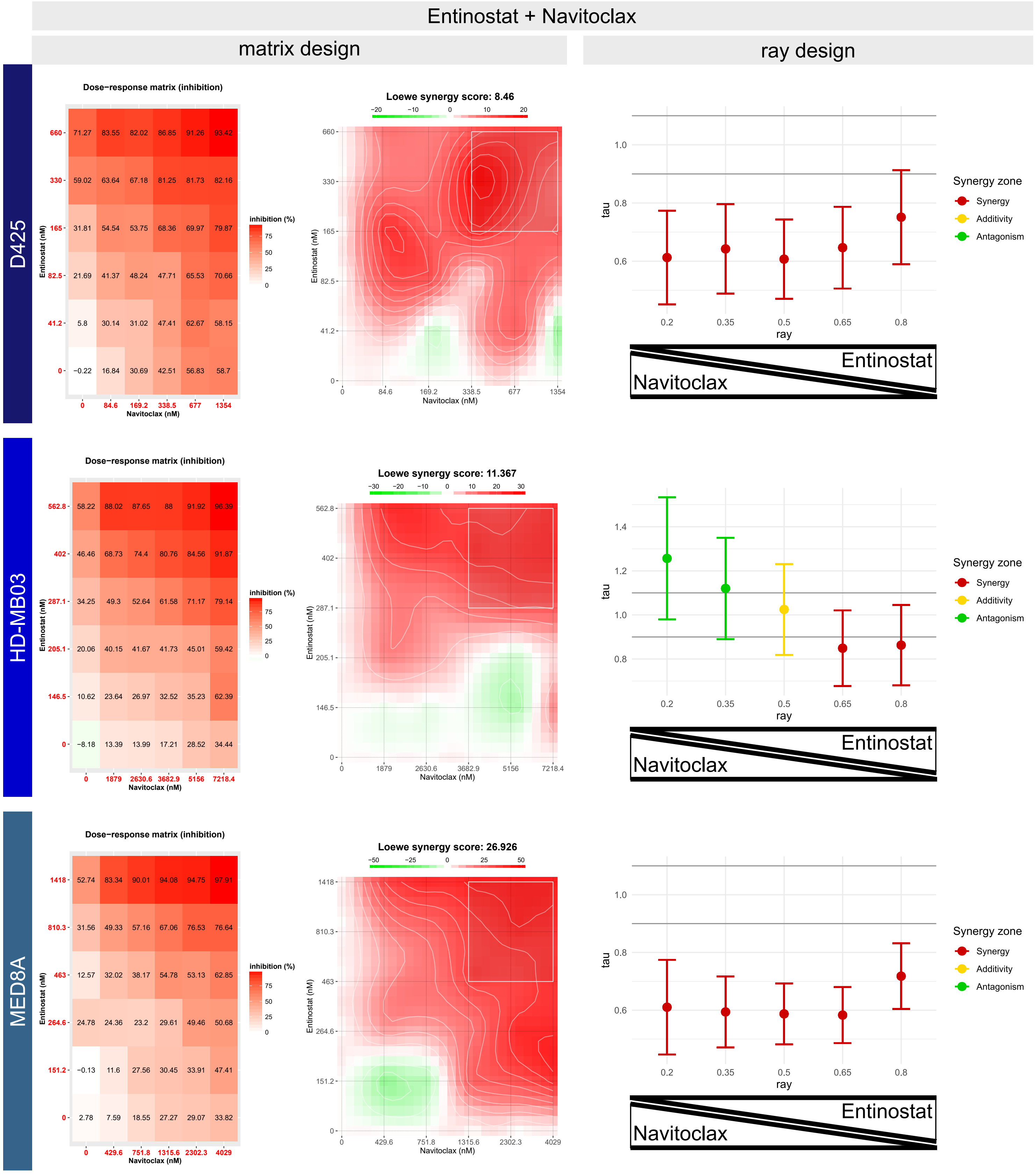

Supplemantary Figure 4

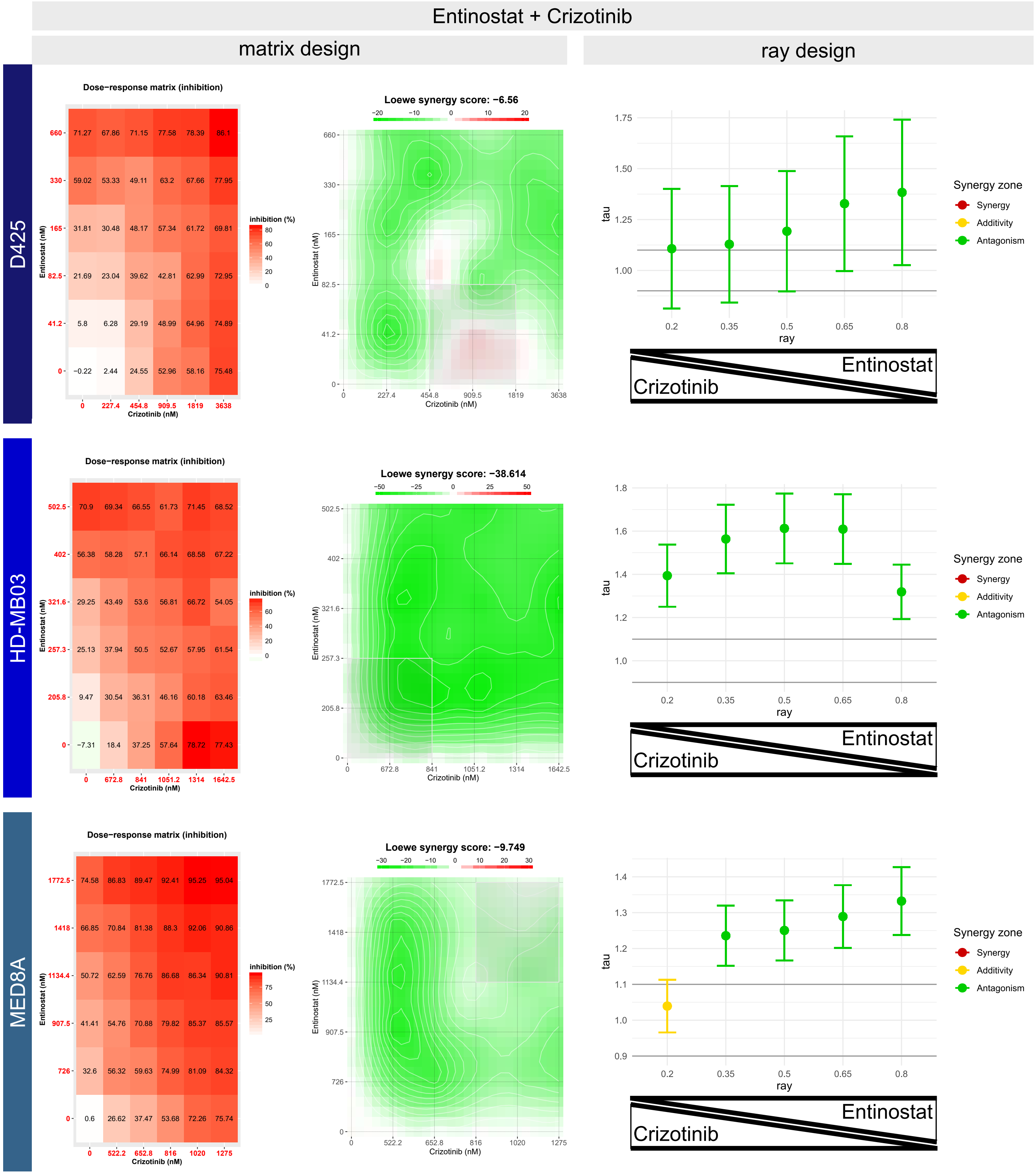

Supplemantary Figure 5

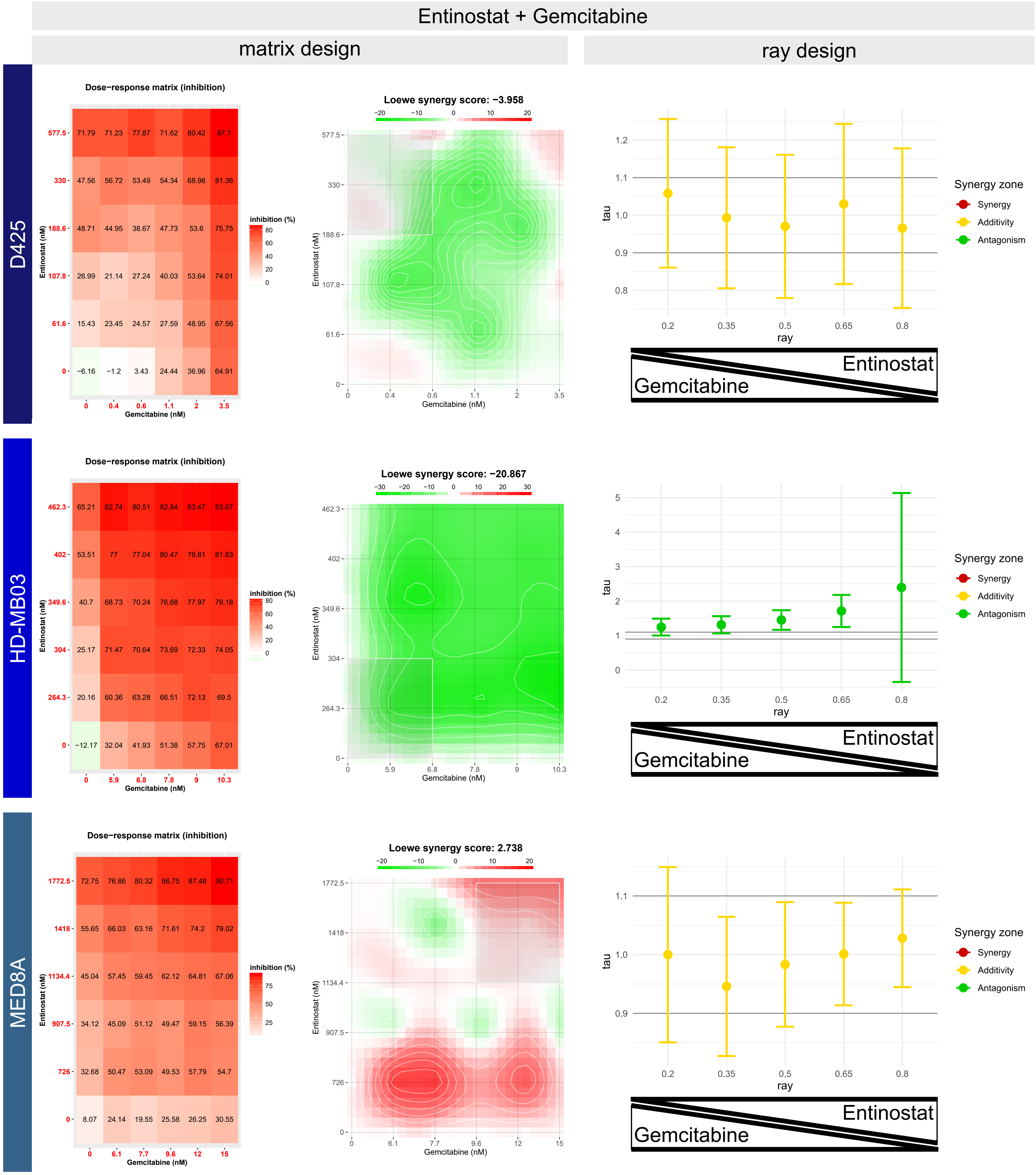

Supplemantary Figure 6

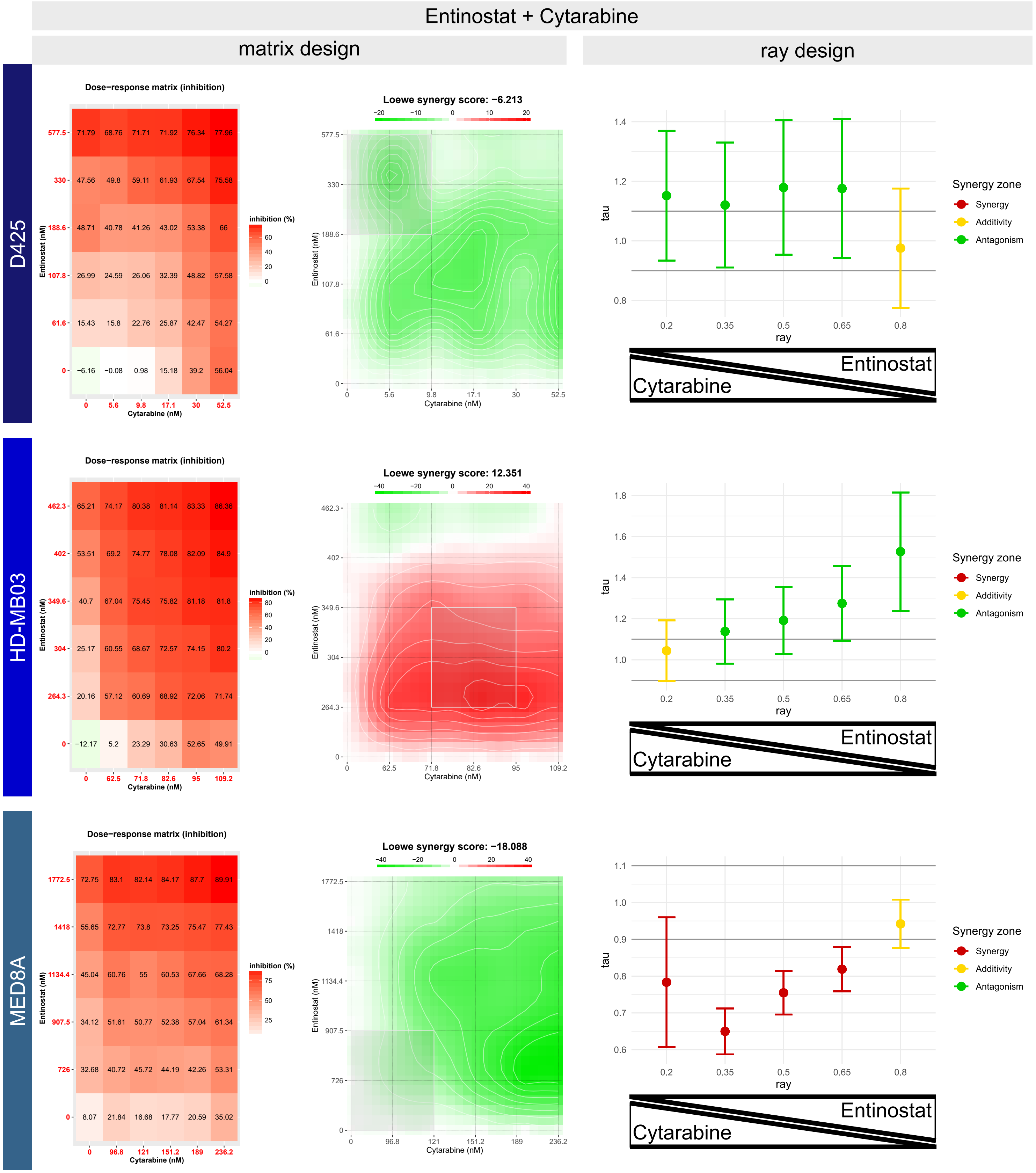

Supplemantary Figure 7

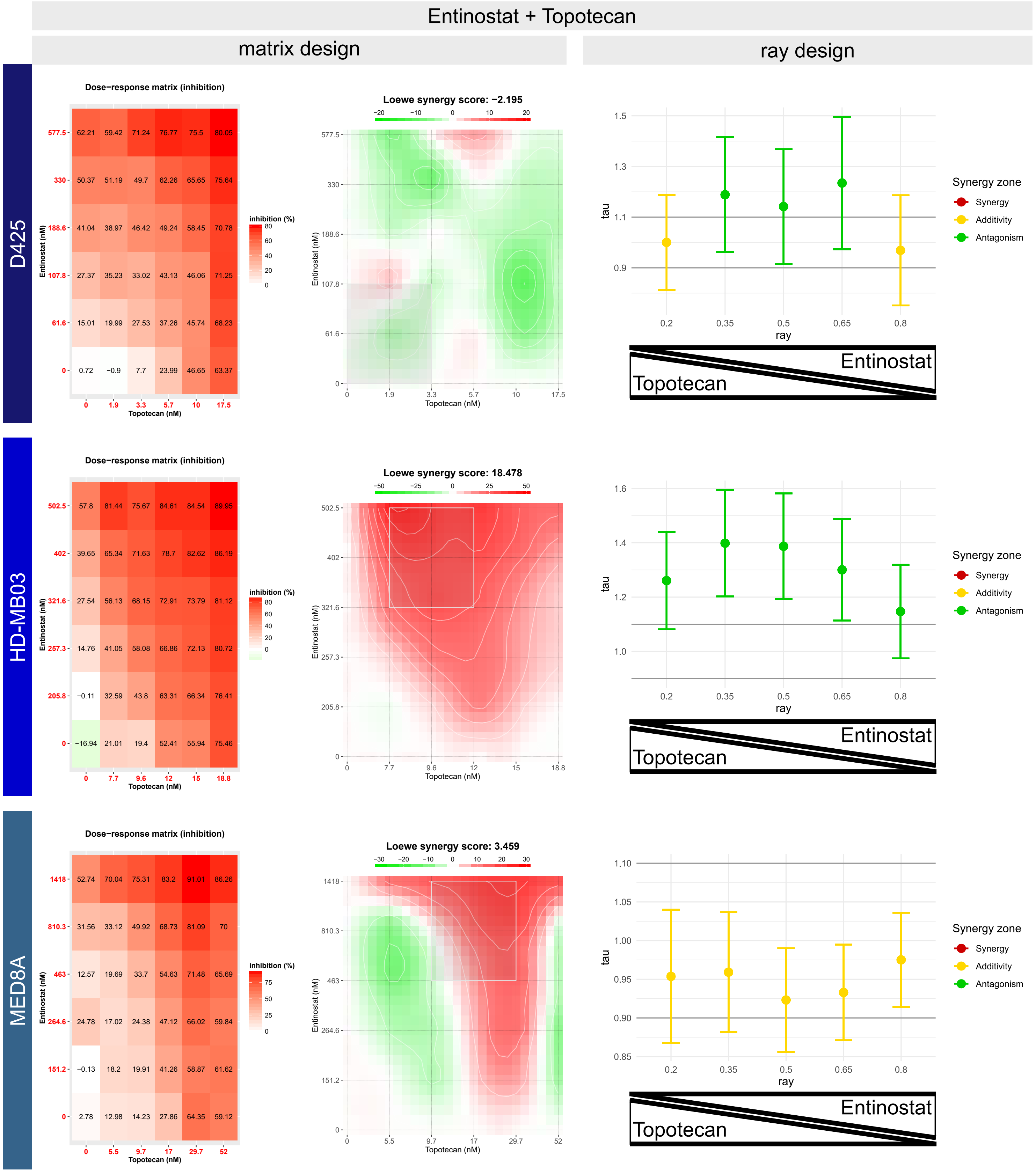

Supplemantary Figure 8

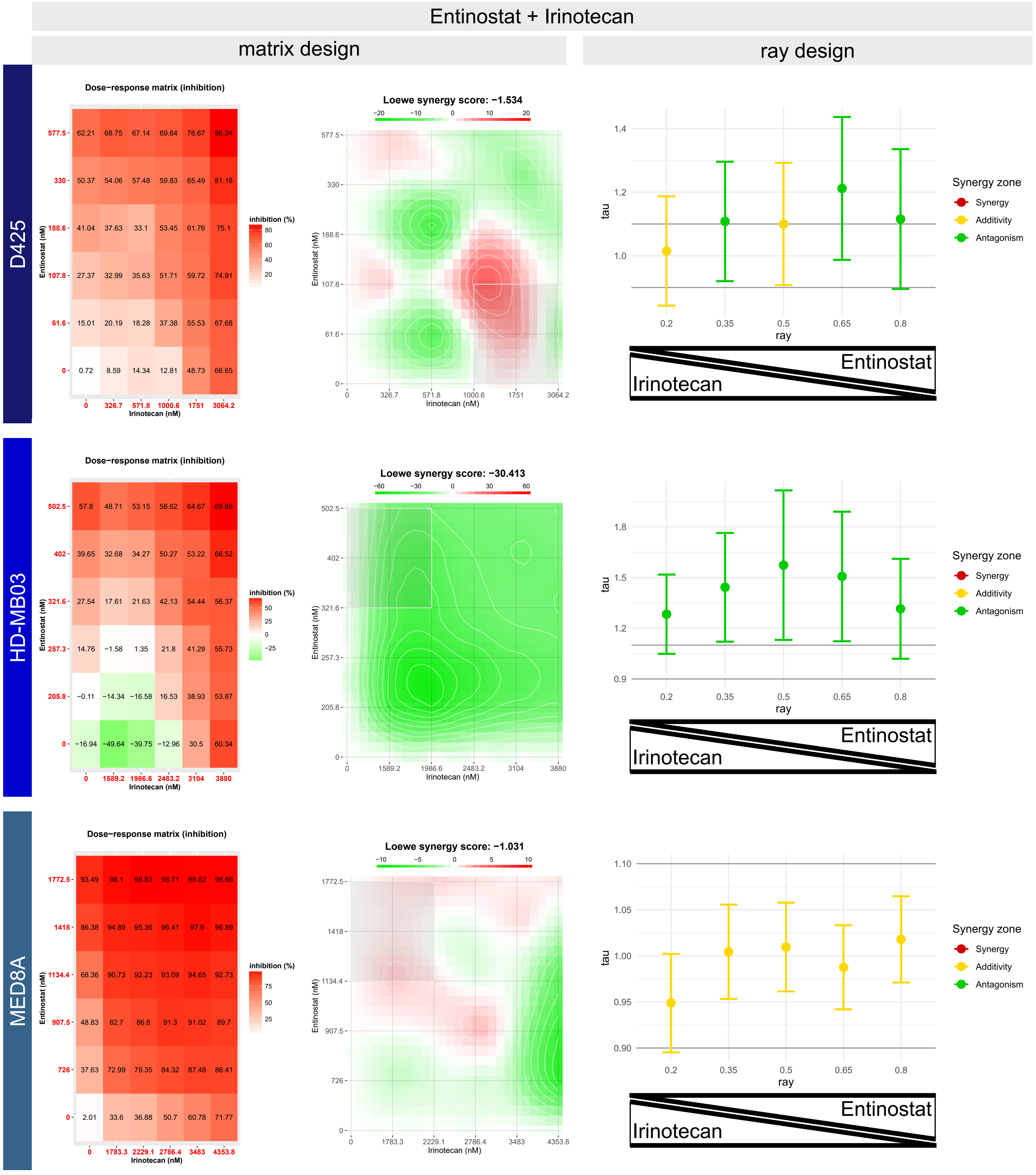

Supplementary Figure 9

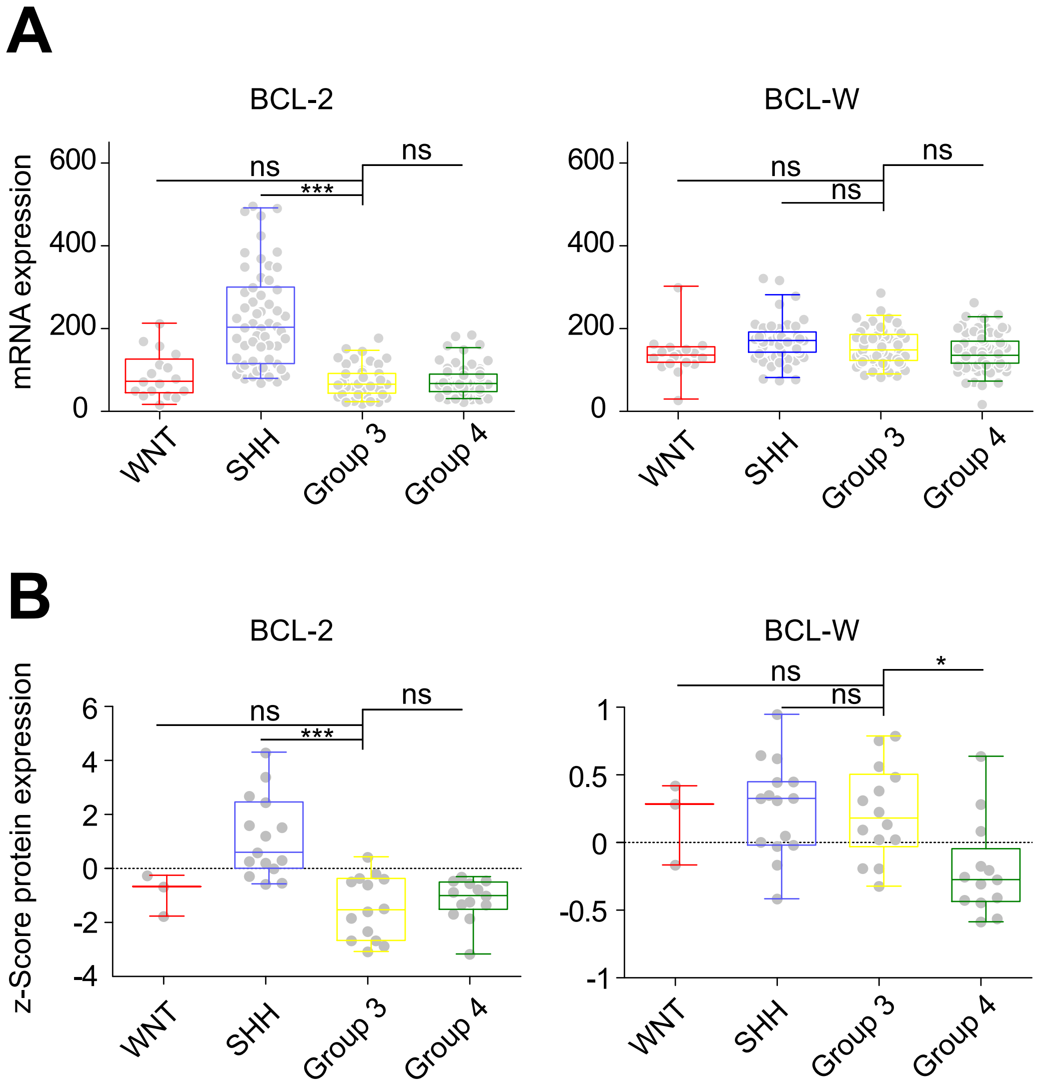

Supplementary Figure 10

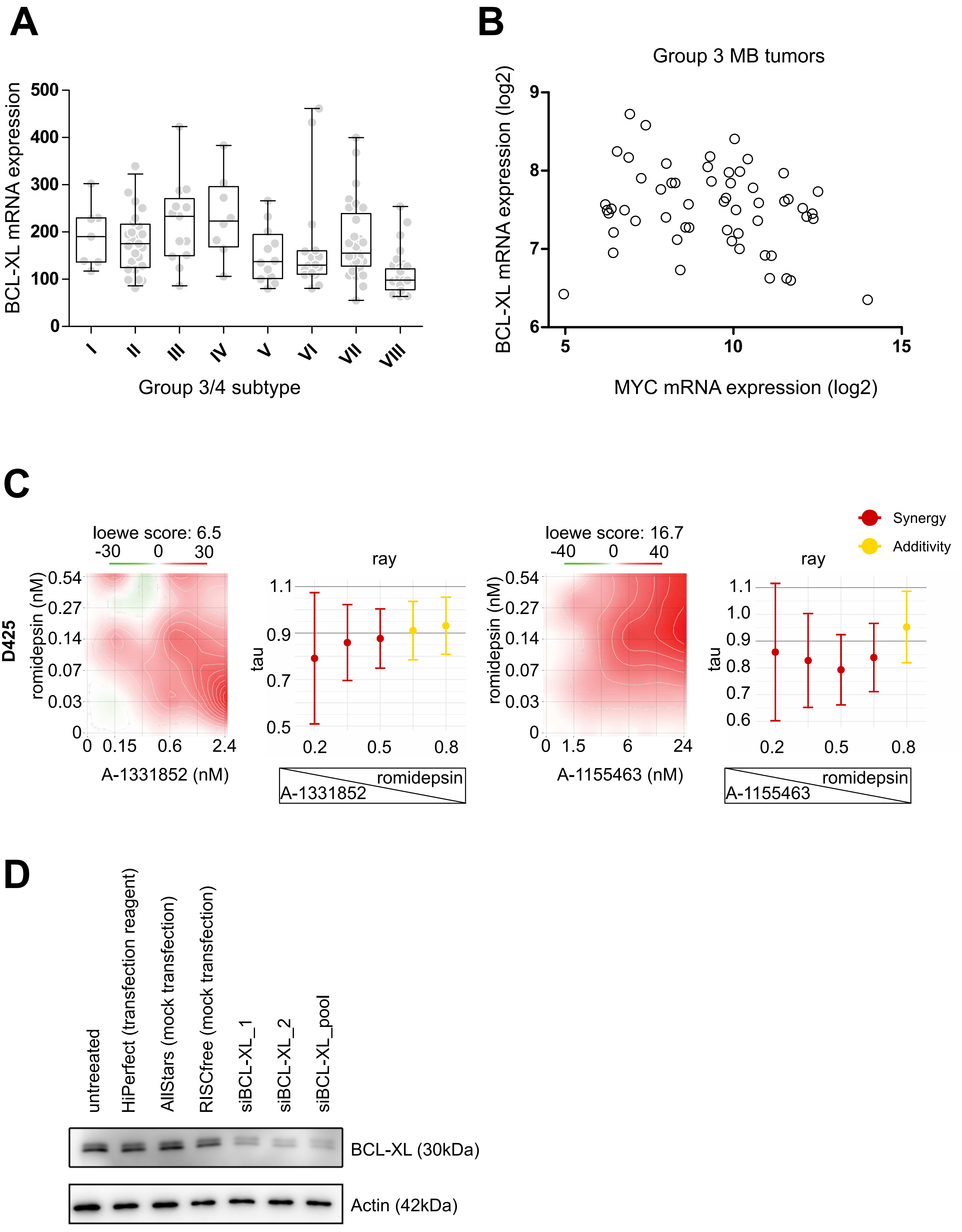

Supplementary Figure 11

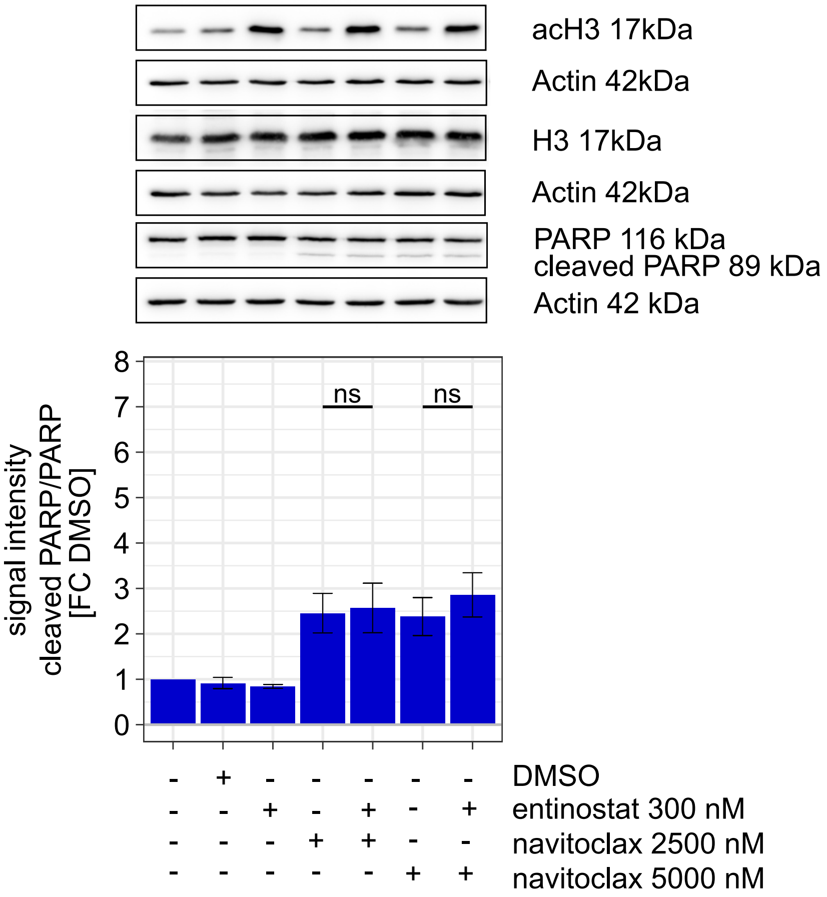

Supplement: Supplementary file 1 — Supplementary file1 (PDF 3254 KB)—Supplementary Figure S1: Single treatment dose response curves of entinostat, navitoclax, crizotinib and gemcitabine. A Single treatment dose response curves of entinostat in D425, HD-MB03, MED8A and UW228-2 cell lines determined by metabolic activity assay. Dark grey line depicts the maximum plasma concentration in patients. Light grey area depicts the concentration range used for the synergy assessments with the 6 top hit drugs. B Single treatment dose response curves of navitoclax, crizotinib, gemcitabine in D425, HD-MB03, MED8A and UW228-2 cell lines determined by metabolic activity assays. Dark grey line depicts the maximum plasma concentration in patients. Light grey area depicts the concentration range used for the synergy assessments with the 6 top hit drugs.Supplementary Figure S2: Single treatment dose response curves of cytarabin, topotecan and irinotecan. A Single treatment dose response curves of cytarabin, topotecan and irinotecan in D425, HD-MB03, MED8A and UW228-2 cell lines determined by metabolic activity assays. Dark grey line depicts the maximum plasma concentration in patients. Light grey area depicts the concentration range used for the synergy assessments with the 6 top hit drugs. B Heatmap of metabolic activity at reported maximal plasma concentration (cmax) in patients. D425, HD-MB03, MED8A and UW228-2 cells were treated with concentrations of entinostat, navitoclax, crizotinib, topotecan, irinotecan, cytarabine and gemcitabine on a 1/2 log distribution. Dose response curves were calculated and metabolic activity at the reported cmax was determined. Supplementary Figure S3: Synergy evaluation of entinostat and navitoclax. Synergy assessment with dose response matrix depicting percentage of inhibition (left), heatmap with loewe synergy score (center) and crossbar plot of ray design (right) of entinostat and navitoclax in three MYC-amplified MB cell lines. Dose-response matrix depicts metabolic inhibition in p [file 11060_2023_4526_MOESM1_ESM.pdf]
